# Analysis and Testing of a Suitable Compatible Electrode’s Material for Continuous Measurement of Glucose Concentration

**DOI:** 10.3390/s20133666

**Published:** 2020-06-30

**Authors:** Nikola Slaninova, Klara Fiedorova, Ali Selamat, Karolina Danisova, Jan Kubicek, Ewaryst Tkacz, Martin Augustynek

**Affiliations:** 1 Department of Cybernetic and Biomedical Engineering, VŠB—Technical University of Ostrava, 17, listopadu 2172/15, 708 00 Ostrava–Poruba, Czech Republic; nikola.slaninova@gmail.com (N.S.); klara.fiedorova@vsb.cz (K.F.); danisovak@centrum.cz (K.D.); 2 Malaysia-Japan International Institute of Technology, Universiti Teknologi Malaysia Kuala Lumpur, Jalan Sultan Yahya Petra, Kuala Lumpur 54100, Malaysia; aselamat@utm.my; 3 Media and Games Center of Excellence (MagicX), Universiti Teknologi Malaysia, Skudai 81310, Malaysia; 4 Department of Biosensors and Processing of Biomedical Signals, Faculty of Biomedical Engineering, Silesian University of Technology, 40 Roosevelt’s Street, 41-800 Zabrze, Poland; ewaryst.tkacz@polsl.pl

**Keywords:** biosensor, electrode, enzyme glucose oxidase

## Abstract

The subject of the submitted work is the proposal of electrodes for the continual measurement of the glucose concentration for the purpose of specifying further hemodynamic parameters. The proposal includes the design of the electronic measuring system, the construction of the electrodes themselves and the functionality of the entire system, verified experimentally using various electrode materials. The proposed circuit works on the basis of micro-ammeter measuring the size of the flowing electric current and the electrochemical measurement method is used for specifying the glucose concentration. The electrode system is comprised of two electrodes embedded in a silicon tube. The solution consists of the measurement with three types of materials, which are verified by using three solutions with a precisely given concentration of glucose in the form of a mixed solution and enzyme glucose oxidase. For the testing of the proposed circuit and the selection of a suitable material, the testing did not take place on measurements in whole blood. For the construction of the electrodes, the three most frequently used materials for the construction of electrodes used in clinical practice for sensing biopotentials, specifically the materials Ag/AgCl, Cu and Au, were used. The performed experiments showed that the material Ag/AgCl, which had the greatest sensitivity for the measurement even without the enzyme, was the most suitable material for the electrode. This conclusion is supported by the performed statistical analysis. On the basis of the testing, we can come to the conclusion that even if the Ag/AgCl electrode appears to be the most suitable, showing high stability, gold-plated electrodes showed stability throughout the measurement similarly to Ag/AgCl electrodes, but did not achieve the same qualities in sensitivity and readability of the measured results.

## 1. Introduction

This publication deals with the proposal of electrodes for the continual measurement of glucose, which consists not only in the construction of the electrodes, but also in the selection of a suitable material for their construction, which measures glucose concentrations using the electrochemical method [1], and comparing the functionality and differences between the investigated electrodes. The research describes the basic theoretical knowledge necessary for proper electrode production and testing.

The measurement of glucose levels from a medical perspective is of prime importance for verifying the health of the patient. Glucose is a natural substance for the organism and thus it is used as an indicator when measuring other hemodynamic parameters. The ideal blood sugar level in the human body is in the range of 3.5 mmol/L to 5.5 mmol/L [2]. There are many methods for measuring glucose in the blood that we can classify according to many perspectives, primarily according to the purpose of their use. The most well-known include the self-monitoring methods, which include laboratory tests, glucometer measuring methods and continuous methods. The foundation of these methods is specific electrochemical or chemical reactions, based on electrochemical photometric methods [3,4]. Electrochemical reactions use specific enzymes for glucose. Enzymes are used to accelerate chemical reactions in glucose evaluation. An enzyme-substrate complex is formed, whereby the reaction results in the starting enzyme and the product. The most widespread method for assessing blood glucose is electrochemical measurement [5]. Glucose oxidase catalyzes the chemical reaction of glucose with oxygen to form gluconic acid and hydrogen peroxide [6]. Hydrogen peroxide is reduced by an electrochemical action to water and free electrodes, resulting in a weak electric current that is proportional to the blood glucose concentration [7].

The research uses a measuring circuit of our own design, which excels in measuring very small electric currents, and an electrode system, for which it is essential to verify the selection of the material for the electrodes. Electrodes from three types of materials were chosen—Ag/AgCl, Cu and Au (Table 1), which were tested using various concentrations of glucose and the enzyme glucose oxidase. Since a narrowly specific electrochemical reaction is used in the work, the material selected for the electrodes must not enter into or otherwise influence the incurred reaction. In the work we verified the hypothesis that Ag/AgCl electrodes measure higher voltages than Cu electrodes and second hypothesis Ag/AgCl electrodes measure higher voltages than Au electrodes. The assumption is that the Ag/AgCl electrodes verified in practice will have greater sensitivity than the other types of chosen electrodes. It follows from this that these electrodes will be able to record more precise values of electric voltage than the other selected materials, which leads to the more precise measured results. The types of materials were chosen on the basis of their various sensitivities and electric characteristics.

The hardware part of the electrode system, its realization and execution are described in detail. A modification of the system with the possibility of data digitization in order to monitor the long-term trend of data over time is also described. The LabVIEW graphical programming environment was used for software implementation and measurement data processing [9]. The measured data were statistically processed in program R and evaluated according to the concentration of the glucose solution used and the electrode material. In each test set, the voltage value detected by the measuring electrode was recorded during the first 150 s without an enzyme and within 220 s using the enzyme glucose oxidase.

The organization of the article is as follows. Section 2 describes the research followed by this paper. The relationship between the papers is described. Section 3 describes the mechanism of the electrode measurement system of which elements it consists. The construction of the electrode part is defined, then the measuring and supply circuit. Section 4 shows the design of hardware for online data recording using a direct connection via a measuring card and also using a microphone element. The materials and dimensions of the used electrodes are also defined in this section. Section 5 assesses the performance of electrodes involving the use of different types of materials at different defined concentrations. Section 6 objectively quantifies the used materials in a concentration-dependent manner using correlation coefficients and other statistical methods. Section 7 discusses the results of the proposed tested electrodes.

## 2. Related Work

Research will continue in the future to develop mini-invasive sensor for the continuous measurement of blood glucose. In order to realize this sensor, it will be necessary to resolve the question of the appropriate immobilization of glucose oxidase to the electrode system and also to choose the most suitable material for the electrode system. This entire electrode system will then need to be shrunk to a sensor that is reduced in size enough to be used for measurement in the dermal interstitial fluid. This chapter outlines current developments and solutions.

Continuous Glucose Monitoring (CGM) has been demonstrated to be clinically valuable and reduces the risks of hypoglycemia and hyperglycemia. This method also improves patient quality of life [10,11]. Continuous glucose monitoring may result in better glycemic control compared to conventional treatment [12,13]. The current trend in patient blood glucose measurement is a 24-h continuous glucose measurement method. The system consists of three parts (sensor, transmitter and receiver) [14]. The electrode is coated with an enzyme layer and inserted into the patient’s subcutaneous tissue. The measurement is based on the electrochemical method as well as self-monitoring glycemia (SMBG) [15]. Blood concentration is a key parameter determining the patient’s health (associated with Diabetes Mellitus). Many methods for the continuous and accurate monitoring of blood glucose have been studied. Electrochemical analysis remains important in this area for its simplicity and quantity. GCM is often neglected due to its invasiveness. Non-invasive methods for estimating blood glucose from tears, saliva and sweat were also studied [16].

Two types of systems are currently available on the market for continuous glucose monitoring. They are a real-time scanning system and an intermittent scanning system. Both systems provide continuous measurement values, but each system has its own unique characteristics, which can be beneficial or negative for individual patients [17,18].

IoT (Internet of Things) health monitoring systems have recently been used to improve health care. However, advanced systems for continuous glucose monitoring using IoT are still in use with many limitations. The feasibility of an invasive continuous glucose monitoring system using the IoT-based approach is currently under study. The IoT-based system architecture from sensor to real-time glucose-presenting back-end system has been designed [19].

Non-invasive methods are desirable to replace the traditional finger prick method because they can facilitate continuous glucose monitoring. The challenges associated with non-invasive glucose monitoring are numerous factors that contribute to inaccurate values. Non-invasive methods of glucose measurement based on: intrinsic glucose properties, blood/tissue properties and breath acetone analysis were tested in recent works [20].

Accurate detection of diabetes and blood glucose is needed for the current needs of improving patient care. Existing systems have shortcomings such as high computational time and low prediction accuracy. For this reason, a diagnostic system was designed that works on the principle of using the machine learning method for the detection of diabetes. The design method was tested on a clinical data set. The method was designed on a filtering method based on a decision tree algorithm. Experimental results have shown that the proposed performance of the system is comparable to previous state-of-the-art methods [21].

For continuous glucose measurements, it is necessary to develop suitable electrodes from a suitable material. A mini-invasive glucose sensor has already been developed with a sensing area at the electrode tip. The electrode is constructed of a platinum-iridium alloy and is embedded in the center of a Polyetheretherketone (PEEK) tube and has been used as a sensing electrode. Glucose oxidase electrodeposition was performed to immobilize the enzymes. The PEEK surface was coated with Ag/AgCl film as a reference electrode. The response of the prepared glucose sensors was tested at a potential of 0.6 V [22].

A reusable, non-invasive and ultrafast high-frequency biosensor was developed based on an optimized manufacturing process of an integrated passive device for quantitative glucose detection. With the aid of the novel biosensor design with hammer-shaped capacitors for carrying out detection, both the resonance frequency and magnitude of reflection coefficient can be applied to map the different glucose levels [23].

Another important chapter is the reduction of the entire biosensor thus allowing the mini-invasive monitoring of glucose in interstitial fluid in the dermal area. Reducing the biosensor size can also lead to the improved stability and reliability of these sensors. The realized device consists of a three-electrode enzyme sensor. The working electrode and counterelectrode consist of platinum. The instrument is capable of dynamically and linearly measuring in-vitro concentrations with sufficient selectivity [24,25].

Another possible chapter in the selection of a suitable material can be the use of nanomaterials or near-zero-index materials. Nanomaterials have become crucial for the development of new technologies in several areas of practical applications. They are often associated only with optics and electromagnetism. Analogous to electrical nanocircuits, the concept of nanoelements of thermal circuits was introduced. Resistors, capacitors and inductors are evaluated in terms of electromagnetic (electrical permittivity) and thermal (conductivity k and convection coefficient) nanostructured properties [26]. Another possibility is to use near-zero-index material. Analytical and numerical results will confirm that the use of near-zero cover materials leads to extraordinary properties in terms of field configurations, low attenuation and bandwidth. The dielectric wire acts as an efficient guide with great potentials for advance nanocircuit and electronics [27].

The research is based on the work of The Proposal of an Electrode for Measuring Glucose Concentration in Blood [28], where the authors presented a basic study of the electrode system for the determination of blood glucose levels. The research suggests an electrode for measuring glucose concentration together with a measuring circuit; this system ensures the supply of the required voltage to the analyzed sample and at the same time detection of current, the magnitude of which corresponds to the electrochemical reaction between the enzyme glucose oxidase and glucose. Due to the enzyme, only glucose molecules are detected in the blood. In order to understand how the electrochemical reaction proceeds and what voltage we detect during the reaction, the measurements take place in two cycles. The first cycle serves to determine how the electrode system behaves during the measurement of pure glucose and how it reacts to different glucose concentrations in the analyzed sample. Based on the results, they were able to distinguish the changes that occurred in the second cycle during the addition of the enzyme. The result confirmed higher voltage values for enzyme samples, where the measured voltage waveform is characterized by a rapid increase in voltage followed by a rapid regression due to the electrochemical reaction and its saturation. The increase of the electrical voltage as well as the concentration of glucose is caused by a chemical reaction that occurs. With the increased concentration of glucose, there are also more ions in the blood that are conductive. Mixing glucose and the given enzyme glucose oxidase results in the creation of water and ions, which increase the conductivity and lower the electric resistance. Two types of electrode systems were used in different designs using the same materials.

## 3. Electrode Measuring System

The electrode measuring system is used to measure the low values of electric current from the electrodes and to convert this electric current into electrical voltage, which is the input value in the chain directly proportional to the value of glucose in the solution. The measurement is based on the electrochemical reaction between the glucose and glucose oxidase, when the electric current resulting from this chemical reaction is recorded and subsequently converted into electrical voltage. The measurement of the solution with the pure glucose was performed for verifying the detected electric current using the measuring circuit with the electrode. To verify the results of the research, each of the electrodes was tested in ten measurements for the individual concentrations and without an added enzyme.

**Figure 1:**
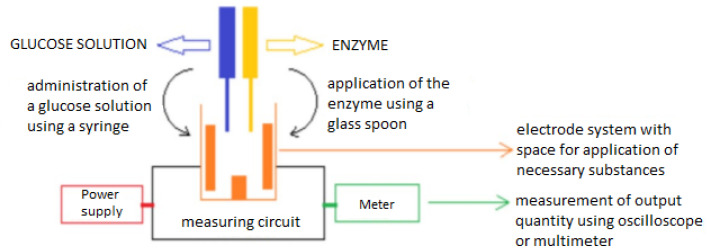
Electrode measuring system.

**Figure 2:**
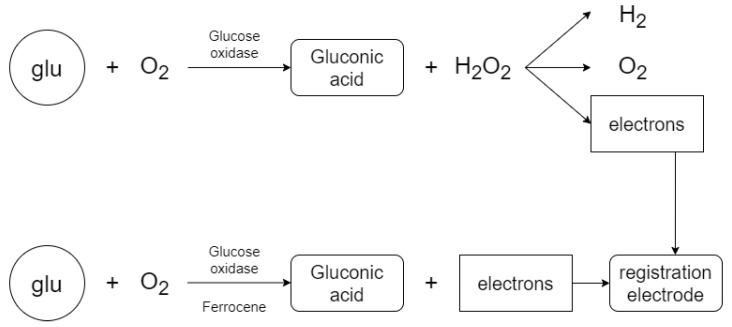
Reactants and chemical reaction products.

The electrode measurement system (Figure 1) in this work consists of three parts—a power circuit, a two electrode system and a measuring circuit. The main idea is the ability to measure glucose concentration in a solution with distilled water (the measurement is performed to verify that the measuring system is capable of detecting electric current) or to measure glucose in a solution that contains the enzyme glucose oxidase [29] to accelerate the catalytic reaction of the glucose (Figure 2). The enzyme glucose oxidase is used for the electrochemical measurement, which catalyzes the chemical reaction of glucose with oxygen. This produces gluconic acid and hydrogen peroxide, which is reduced by electrochemical action to water and free electrons. This produces a small electric current which is proportional to the glucose concentration in the solution. Measurements are usually distorted by the involvement of oxygen in the reaction (formation of hydrogen peroxide). The method was modified with the ferrocene mediator reagent, which reduces the catalysis of hydrogen peroxide [30,31,32].

The glucose oxidase enzyme in the solution causes the measuring system to measure a higher current value during the biochemical reaction than when the enzyme is not in the solution. The resulting signal, which is measured, is proportional to the glucose concentration in the sample solution, and is generated by the movement of free ions. Ions of different kinds are present in the solution and therefore it is important to select only glucose molecules (using an enzyme). The enzyme in the solution causes a chemical reaction to form gluconic acid and hydrogen peroxide, which is further cleaved to hydrogen, free ions and oxygen to increase the resulting current. The electrical potential in the solution must be 0.6 to 0.69 volts for decomposition. The electrical current generated by the chemical reaction is detected by the electrode system and further converted to an electrical voltage from which the resulting glucose concentration is evaluated more accurately [30,31,32].

### 3.1. Construction of the Electrode Part

It was necessary to design an electrode construction that would have the suitable sensitivity and stability to record the measured results.

**Figure 3:**
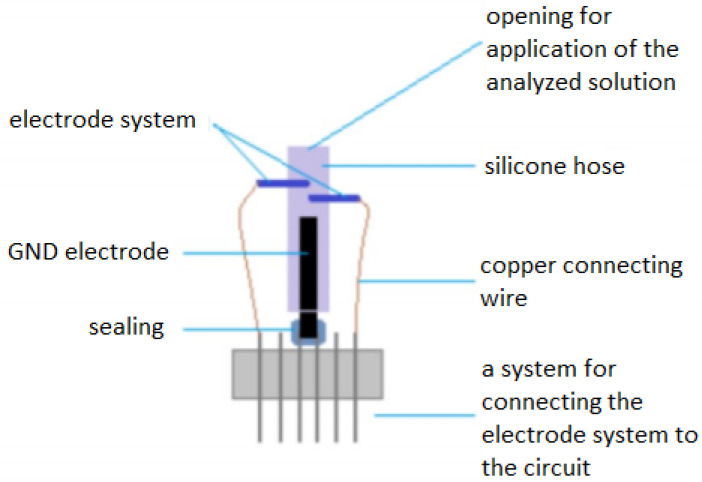
Construction of the electrode.

The main element of the electrode part is a set of electrodes (Figure 3), which are designed as a pair of small rollers interconnected by copper connecting wires with pinheads and a supply and measuring circuit. Each of the electrodes has a different function in the system, which is given by connecting the wires to the circuit. The electrodes are mounted in a silicone tube.

The electrodes are designed as a metal cylinder with a length of 4 mm and a cross-section of 0.5 mm. The electrode gap is 0.97 mm. One electrode is under voltage of 0.6 to 0.69 volts (hydrogen peroxide decomposition). This electrode is connected to the circuit and is marked as working. The dimensions of the electrode were chosen experimentally, due to the available technologies and possibilities. The intention was to create an electrode with the smallest possible dimensions. The design of the electrode dimensions was limited by the available materials. The size of the electrode was not crucial for this research, as it is a prototype electrode. Appropriately chosen size of the electrode system (Figure 3) affects the whole results of the measured electrical voltage. In the initial measurements, it was verified that if the electrode pads are closer together, the electrode measures in a narrower range. This effect is desirable especially when reading the concentration of glucose from the blood, when due to the narrower range, even less different concentrations will be recognizable.

Decomposition occurs when hydrogen peroxide is cleaved. Free ions move to the second electrode (metal roller), the measuring electrode, which registers it. The processing then takes place by transferring the measured small current to the measuring circuit, where it is converted to electrical voltage.

The silicone used to connect all the components of the electrode system and to apply the test solution is electrically non-conductive and does not affect the proper functioning of the system or the chemical reaction of the glucose enzyme. The silicone tubing has a diameter of 4 mm and the space for application of the test solution is $25.13\times 10^{-8}/L$. The tubing has two electrode placement holes at the top, and the bottom end is sealed with a grounding electrode (to eliminate interference with the measurement system).

The grounding electrode is constructed as a copper wire system in the insulator. The terminal parts are connected with two pinheads to the circuit. A mixture of synthetic resins, paraffins and copolymer was used to seal the electrode. Connecting wires connect gold-plated electrodes and pinheads. One conducts voltage to the working electrode to decompose hydrogen peroxide and the other conducts the detected signal to the circuit from the measuring electrode. Pinheads are in two rows with six outlets and are intended to provide a connection to the measuring circuit.

### 3.2. Measuring and Power Supply Circuit

For the proper functioning of the system, it is necessary to construct a power supply circuit and a circuit to perform the measurements. The entire system is comprised of resistors with various resistance values and four opamps.

**Figure 4:**
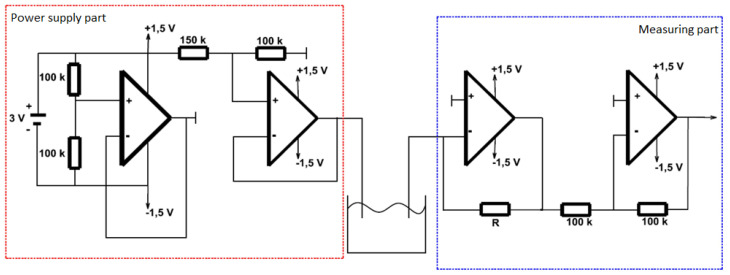
Diagram of power supply part and measuring part.

The power circuit (Figure 4) is designed to adjust the incoming voltage to 0.6 volts for the working electrode and then to process the converted voltage from the measuring electrode and measure it using an oscilloscope. The power supply circuit is supplied with a DC voltage of 3 V (two AA batteries in series). The voltage divider is reduced to 1.5 V (using two 100 k Ω resistors). This voltage is then routed to the positive input of the operational amplifier, which monitors the voltage values. The voltage at the input and output of the operational amplifier is therefore the same. An artificial ground is created from the output of the operational amplifier. Another voltage divider with 150 k Ω and 100 k Ω resistors creates a voltage of 0.6 V, which is applied to the working electrode by means of a connecting wire and pinhead [15,28,30,33].

The measuring part (Figure 4) of the circuit is connected to the measuring electrode. The first operational amplifier in this section converts the current to voltage (the output voltage depends on the value of the resistance trimmer, the resistance of the solution with the submerged electrodes, and the input voltage of the working electrode). A negative voltage drop is output from this opamp. The last opamp adjusts the negative voltage, it serves only as a signal inventor. The resulting positive voltage at the end of the measurement chain then displays a voltage value proportional to the glucose concentration in the measured solution [15,28,30,33].

The scope of the measuring circuit is limited by the supply voltage of the electronic microammeter. The sensitivity of the circuit is limited by instabilities, such as surface phenomena on the electrodes or the noise of the electronic circuits.

## 4. Design and Implementation of Hardware Chain for Online Recording

**Figure 5:**
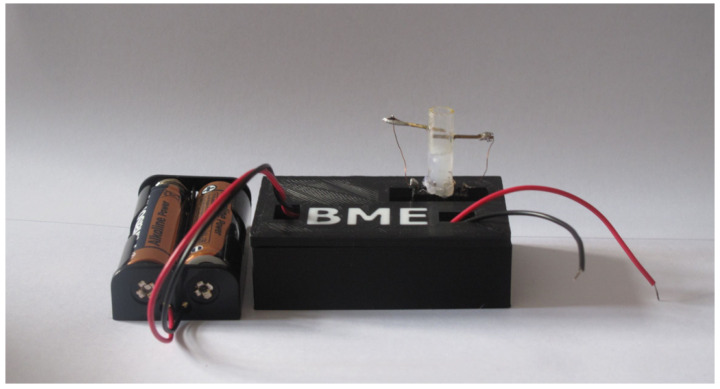
Complete measuring system.

The main task of this work was to create a complete measuring and imaging electrode system (Figure 5) able to monitor the value of the resulting voltage corresponding to the glucose concentration. In the previous work, it was found that the generated analog signal is a small DC voltage, which can be processed in several ways. The LabVIEW graphical programming environment (Laboratory Virtual Instruments Engineering Workbench) [34] was used to design and implement the software application for processing the measured data. The DAQ (Data Acquisition) card is used to connect the measuring and power supply circuits to a computer interface and to convert the measured data online into digital form. The DAQ Data Acquisition Card is used for a direct connection. The NI USB 6009 measuring card (National Instruments) was used for measurements in this work. The card has eight analog inputs (14-bits), two analog outputs (12-bits), twelve two-way digital lines and one 32-bit counter. The counter has a maximum input frequency of 5 MHz and an input high voltage of 2.0 V. Communication with the DAQ card was performed with the use of the DAQmx library in the LabVIEW program [35].

### 4.1. Microphone Input Element for Computer Input

**Figure 6:**
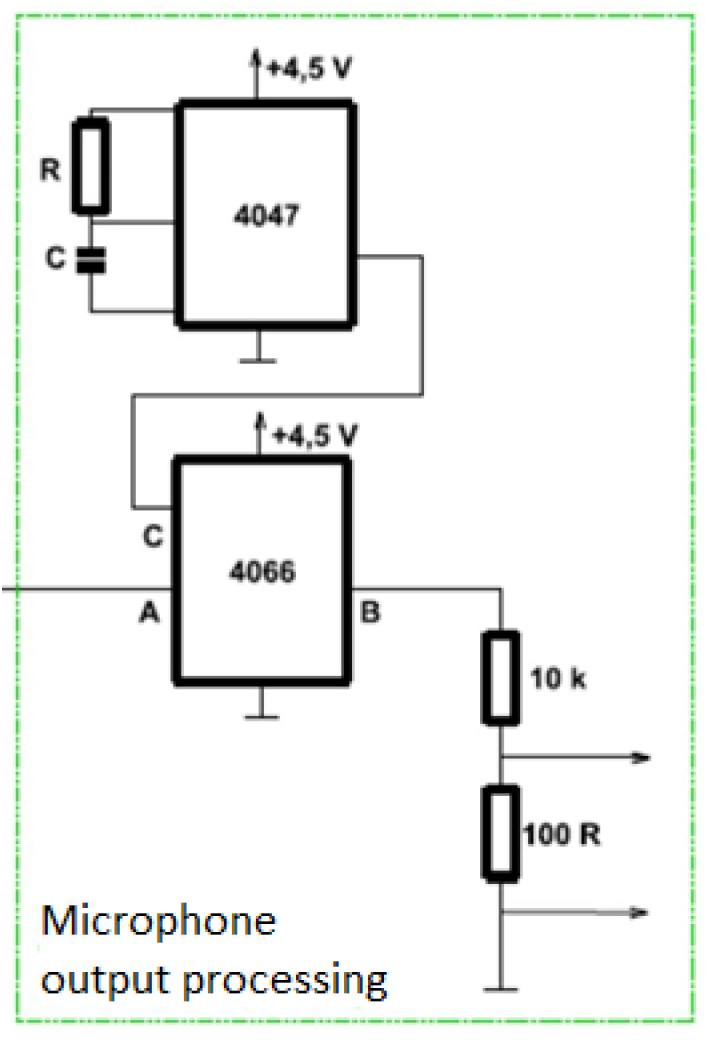
Diagram of microphone output processing.

An alternative to the measuring DAQ card is the connection through a microphone input. The proposed converter must be able to process a small direct signal and convert it into the output of the converter which can then lead into the microphone input using a cable with a jack. Here it is then converted into a digital signal using an A/D converter. To use the computer’s microphone input (Figure 6), it is necessary to obtain AC voltage at the output of the converter, which is converted to a digital signal by the A/D converter. When designing the conversion element, we have to consider the microphone input sensitivity, which is 100 mV. The first circuit utilized in this section is the Texas Instruments CMOS Low-Power Monostable/Astable Multivibrator CD4047B circuit operating in a monostable or astable mode. The astable state, when the circuit periodically flips from one state to another, was used for this work. The second circuit 4066 is a low resistance analog switch comprised of four independent double-sided switches. This circuit is used to generate a DC pulse signal. The resulting circuit with the conversion element conducts a signal from the inverter output to the Texas instruments Quadruple bilateral analog switch SN74HC4066 circuit. If the CD4047B circuit turns on logic 1, the input from A is coupled to output B. The output voltage is reduced by a 10 k Ω resistor divider and 100 Ω and then enter the microphone input. A disadvantage of using the analogue microphone input can be the limited non-linearity and pre-amplifier noise.

### 4.2. Types of Electrodes

The definition and selection of the proper material for the electrodes was essential for this research. The material must show the highest stability and resolution when measuring in the designed measuring circuit. That is why three types of materials that are used in medical practice were used. The characteristics of the electrode material are given primarily by the external layer of the electron shell of the element atoms. When approaching the atoms and electrons from the outer layers, there is an interaction and creation of a bond. The characteristic of the material is defined by the arrangement of the atoms that make up these bonds. A metallic bond is comprised of a structure of positive ions, located systematically in a crystal lattice, surrounded by valence electrons entering between the atoms. The presence of these free electrons causes the thermal and electrical conductivity of the metals. Conductive materials can be divided into two categories, materials with high electrical conductivity and materials with high electrical resistance. Materials with a high electrical conductivity were used in this research. The materials selected for this research are characterized by a high electrical and thermal conductivity (mainly silver and copper). Gold-plated electrodes, on the other hand, have a very high chemical resistance.

**Figure 7:**
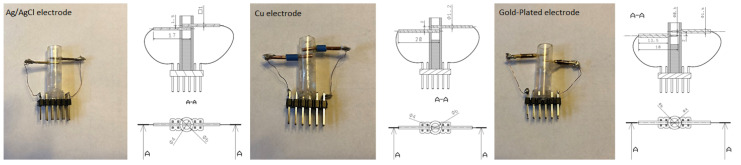
Types of electrodes used in this work.

The electrode system was tested on three materials (Figure 7) (Ag/AgCl, Cu and Gold-plated), but primarily on gold-plated and copper wires. Identical dimensions have been chosen for the materials for the easier processing and evaluation of measurement results.

The first electrode formed was an Argentine chloride electrode formed from the ECG (Electro Cardio Graph) of the limb electrodes. This material was also chosen because of the most widely used material for the construction of the sensing electrodes, since it has the lowest polarization potential. Structurally it is a silver wire covered with a layer of silver chloride with a square cross-section with a length of 1 mm. The silicone tubing and thin copper wire diameter are the same for all electrode materials. The solution application area is the same for all electrodes, 0.2 mL.

The second electrode is made of copper with a diameter of 1.2 mm and a distance of 2 mm. Technical copper is a metal that has the second highest electrical and thermal conductivity. The copper material has a high electrical conductivity, but insufficient chemical stability.

The third gold-plated electrode has a diameter of 0.6 mm, making it the thinnest of all electrodes. Parts of the electrodes outside the measuring cell have a 1 mm larger diameter, which prevents leakage of the measured solution from the silicone tube. Gold-plated electrodes are often used in medical diagnostics as gold has one of the highest degrees of chemical resistance.

## 5. Functionality of Electrodes Including Use of Various Materials

The functionality of the electrodes was tested on the created software with experimental measurements, where the voltage values from the measuring system were determined. These values were recorded every 1 s for 150 s. Each material was tested for four different glucose concentrations and each individual concentration was tested ten times with the enzyme and ten times without the glucose oxidase enzyme. The enzyme measurement was extended to 220 s due to the long onset of the chemical reaction. For testing, a powdered enzyme (VWR) was used, which was applied in an amount of 0.01 g to the solution after 20 s of ongoing measurement. The molar concentration defines the amount of glucose dissolved in the sample to be examined. This molar concentration is directly proportional to the amount of the substance (mole) and inversely proportional to the volume of the solution (in liters). To obtain the desired solution of a preselected concentration, it is necessary to calculate the amount of glucose (1 mmol equals 0.18 g), which must be mixed with distilled water (for the experiment technical distilled water with electrical conductivity of 0.0015 Sm^−1^ was used). The temperature of the solution must also be taken into account as the conductivity increases with increasing temperature. Four glucose concentrations (6 mmol/L, 12 mmol/L, 20 mmol/L and 25 mmol/L) were used for experimental measurements. These solutions were chosen because it was not possible to perform the testing on whole blood. Before each measurement, the electrodes had to be rinsed with distilled water [36].

### Comparison of the Results of Measured Materials

**Figure 8:**
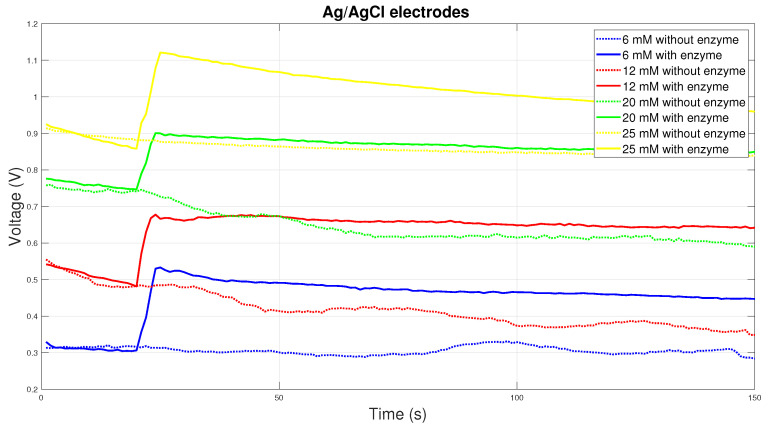
Voltage comparison between Ag/AgCl electrodes.

Ag/AgCl electrodes are very often used in medical diagnostics. Therefore, this material was chosen for testing. The Figure 8 shows the course of the electric voltage over time, where there is a sharp increase in the electric voltage of the electrode with the enzyme due to the electrochemical reaction and subsequently the relatively constant course of the electric voltage. Here, the electrochemical reaction and the material used may be unsuitable. Glucose is consumed here, so it is not possible to record real measured values.

**Figure 9:**
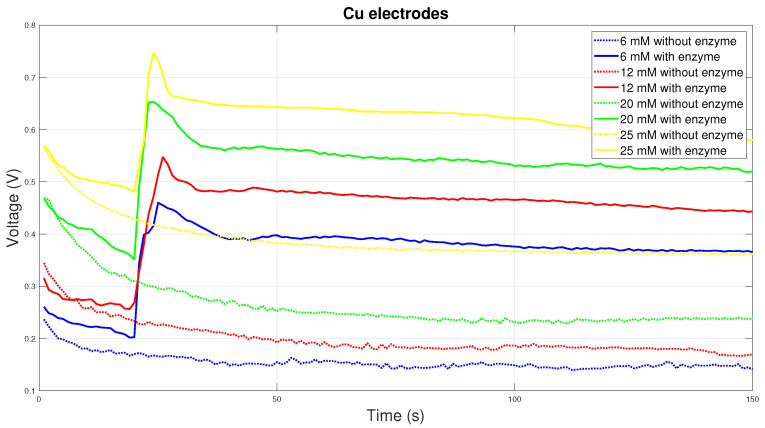
Voltage comparison between Cu electrodes.

Copper electrodes have high electrical conductivity, but do not have the necessary chemical stability, so they are not the most suitable for measuring blood glucose. The graph in Figure 9 shows the course of measurements on copper electrodes. For electrodes with enzyme, there is a sharp increase and subsequent decrease in electrical voltage. The measured values then gradually decrease over time. The measured initial voltage peak is the result of an improper chemical reaction.

**Figure 10:**
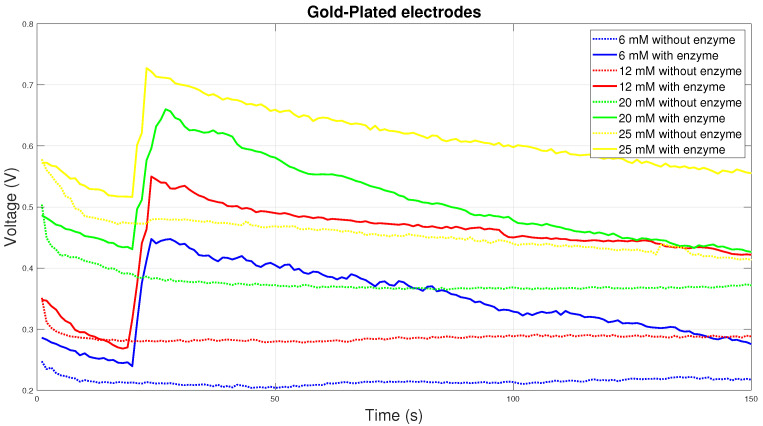
Voltage comparison between Gold-Plated electrodes.

Although gold-plated electrodes do not show the best electrical conductivity, on the contrary, they are the most chemically stable. For this reason, they are most often used to measure blood glucose in Diabetes mellitus. The graph in Figure 10 shows the course of the chemical reaction at the gold-plated electrodes. From the graph it can be concluded that the reaction with the enzyme is the most stable at all concentrations. Initially, there will be a sharp rise in electrical voltage with a gradual decrease over time.

**Figure 11:**
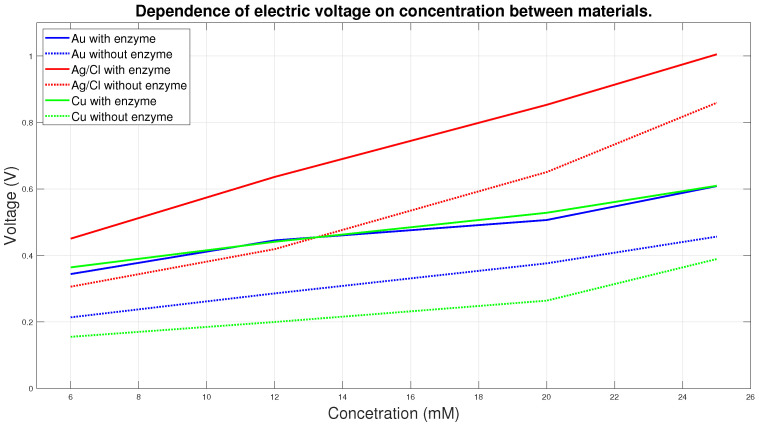
Dependence of electric voltage on concentration between materials.

The graph in Figure 11 shows the course of 6 curves, where each curve shows the dependence of the electrical voltage on the concentration. Solid lines correspond to measurements with enzyme, dashed lines to measurements without enzyme. Values for individual concentrations were averaged from all measurements for individual concentrations of 6, 12, 20 and 25 mM. It is clear from the graph that the voltage increases in direct dependence on the increasing concentration. Measurement without the use of enzyme does not provide relevant information, as the necessary electrochemical reaction does not take place here. From the curves shown, it can be clearly seen that the Ag/AgCl electrodes are the most sensitive, in all embodiments the measurements measure the highest electrical voltage and in the measurements with the enzyme the dependences are almost linear. Measurements with Au and Cu electrodes measure at lower values of electrical voltage and the differences between these measurements are not large. Based on our specific measurement, Ag/AgCl electrodes are the most suitable for the proposed system in terms of electrical conductivity.

## 6. Results

The results of the individual materials are evaluated depending on the results of the measurements in various concentrations, with and without the use of the enzyme while recording the value of the voltage of the voltage after 1 second. Technical distilled water with a specific electrical conductivity of 0.0015 $S.m^{-1}$ was used for the experiment. The pure distilled water was measured before each measurement so that it was possible to define the sufficiently washed measurement space. The stability of the measurement and linearity in the given time interval, the dependency of electrical voltage over time, was primarily evaluated on the measured date from the electrodes.

Within each measurement, voltage values were recorded over 150 s for the enzyme-free concentration and 220 s for the enzyme measurement. The testing with the enzyme took place over a longer time due to the short change of the voltage after the considerable increase during the chemical reaction. It was assumed that the value of the measured voltage will increase with increasing concentrations. This assumption has been verified for each electrode separately and confirmed. Further, correlation analysis was used, comparing the strength of the linear dependence of tested data (Pearson correlation coefficient used).

The highest correlation between the voltage and the concentration of the solution was demonstrated with the Ag/AgCl electrodes (Table 2) using the enzyme. The resulting Cu electrodes and Au electrodes had comparable results (Table 3 and Table 4). The results confirmed that there is no linear dependence between the solution concentration and the measured voltage.

The following hypotheses were chosen for further statistical processing according to its electrical properties (conductivity). The hypotheses were determined from the properties of selected tested materials:

Ag/AgCl electrodes measure higher voltages than Cu electrodes.
Ag/AgCl electrodes measure higher voltages than Au electrodes.
Au electrodes measure higher voltages than Cu electrodes.

The hypothesis “Au electrodes measure higher voltages than Cu electrodes” was chosen on the basis of higher chemical stability of Au than Cu. In terms of electrical properties, Au has a lower conductivity (and greater resistivity) than Cu.

To test the normal distribution of data, the Lilliefors normality test (Table 5, Table 6, Table 7 and Table 8) was used to confirm the normality with the hypotheses:

H0 (null hypothesis): data have a normal distribution.
H1 (alternative hypothesis): rejection of H0.

The decision on the null hypothesis was resolved on the basis of *p*-values for individual measurement sets. All these *p*-values were greater than 0.05 and therefore we do not reject the null hypothesis about data normality at the 5% significance level.

To verify the analysis of variance, the Bartlett test (Table 9) was used with the hypothesis:

H0: $\sigma_{1}^{2}=\sigma_{2}^{2}=\sigma_{k}^{2}$.
H1: rejection of H0.

In all cases, both with and without the enzyme, the resulting *p*-value was greater than 0.05, and therefore we do not reject the null hypothesis about the variance of the 5% significance level and the variance analysis test can be applied.

Box graphs were generated for all concentrations with and without the enzyme measurements. The horizontal axis shows the individual types of electrode materials, the vertical axis determines the voltage. There are no large variations in the box graphs. It was also evident from the graphs that the Ag/AgCl electrodes showed the most stable and highest measured voltage values. The next step was to compare which electrodes measure higher voltages and by how much, which was done using the Tukey test.

The post-hoc analysis hypotheses were formulated as follows:

H0: mean values in pairs are: Ag/AgCl-Au, Au-Cu, Ag/AgCl-Cu.
H1: mean values in pairs do not equal.

In the non-enzyme test results, in all cases, the *p*-value is less than 0.05, and therefore the null hypothesis is rejected and it can be said that the differences between the voltage values of the individual materials are statistically significant. In the case of enzyme testing, the *p*-values for all material pairs are less than 0.05 and therefore the difference between the measured values can be considered to be statistically significant. The results were statistically significant in all cases.

For enzyme-free measurement results, the results could be interpreted as follows: Ag/AgCl electrodes measured higher voltages than copper or gold-plated electrodes. Gold-plated electrodes, in turn, measured higher voltage values than copper electrodes. The enzyme results were interpreted the same way. Ag/AgCl electrodes measured higher voltages than gold and copper electrodes. Gold-plated electrodes measured higher voltages than copper electrodes. The conclusion from the testing is therefore that the differences between the electrode materials are statistically significant.

**Figure 12:**
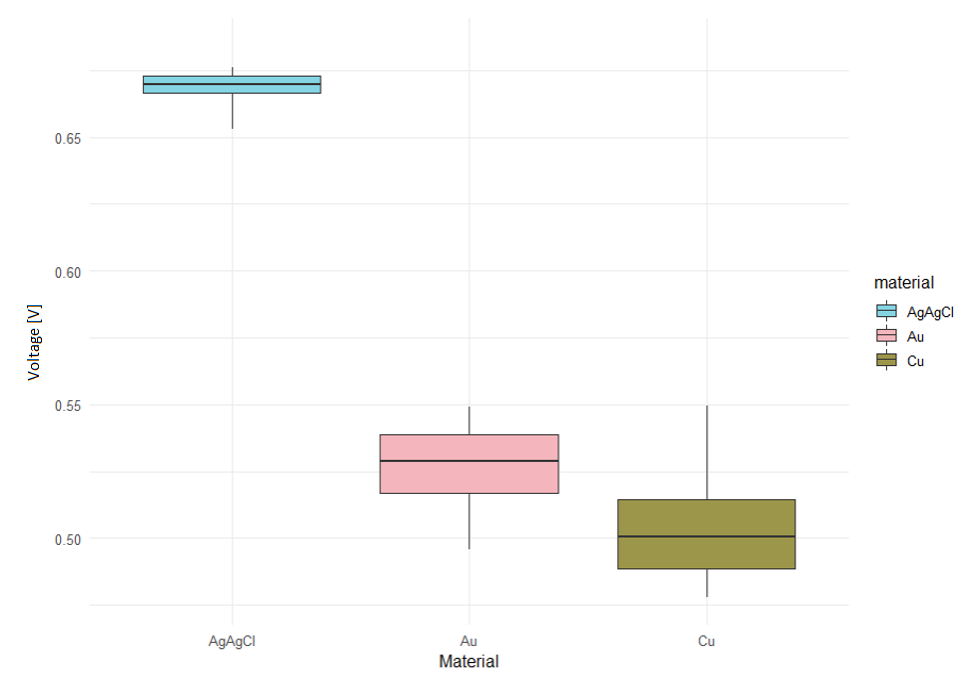
Voltage in concentration of 12 mM with enzyme.

The box graph of the measured voltage at a concentration of 12 mM with the enzyme (Figure 12) confirmed our hypothesis that the Ag/AgCl material will have the smallest dispersion of the measured voltage. This type of electrode is thus capable of recording more precise measured values that the types Au or Cu.

## 7. Discussion

The aim of the work was to create a complex measuring electrode system with the ability to record voltage into a computer, in reference to the glucose concentration in a sample. Three types of electrodes, argent chloride, copper and gold-plated, were tested during the measurement.

The appearance of the individual voltage waveforms between measurement sets with different electrode materials was very different. If values were rounded to one decimal place (sufficient for glucose measurements), the values in the individual sets would be very similar. The values measured with the Ag/AgCl electrodes are significantly higher for all measurements than the values measured with other electrodes. Greater voltage differences were observed between the waveforms. The enzyme-free solution had a slowly descending curve, while with the enzyme, the curves had a sharp increase in tension and then a slow decrease. Enzyme measurements showed more stable voltage values. Thus, these electrodes also had the narrowest scope of the measured voltage, therefore it was possible to detect more precise measured values with them.

The copper electrodes had a lower voltage than the Ag/AgCl electrodes. In the enzyme-free measurement, the moment of application of the solution was visible (a higher voltage values recorded). When the enzyme was applied, the curves had a sharply decreasing character after reaching the highest value. Copper electrodes have common, more stable values after the application of the enzyme with the Ag/AgCl electrodes.

The gold-plated electrodes in the enzyme-free measurement had noticeably higher voltages when applied to the solution than copper electrodes. Thus, the voltage decreased more rapidly. Enzyme values were not more stable than non-enzyme electrodes.

Ag/AgCl electrodes were chosen as the most suitable material for measuring the glucose concentration in the solution. These electrodes measured the highest values of all electrodes and, at the same time, showed the greatest voltage difference at individual concentrations. Measurements using Ag/AgCl electrodes also showed the greatest stability (the curves behaved steadily). The question remains whether this type of electrode would withstand continuous measurement in terms of material stability. This point will be the subject of further research. All claims were verified statistically. The analysis also found that the relationship between the glucose concentration of the solution and the measured voltage is not linear. On the basis of the testing, it was discovered that even though the Ag/AgCl electrode appears to be more stable with the most precisely measured values, gold-plated electrodes show stability during the course of the measuring similar to Ag/AgCl electrodes, but they did not reach the same quality in the resolution. Gold-plated electrodes were chosen as the most suitable due to the high chemical stability of the material. They are also one of the most commonly used materials for the production of electrodes for measuring blood glucose.

The future development of the work will be directed towards the research and realization of the immobilization of the enzyme glucose oxidase on the electrode. Continuity testing and the incorporation of an electrode measuring system and immobilized enzyme into a catheter suitable for medical applications will also be performed. Following the successful selection of the immobilization method, the entire proposed system will be optimized and tested on a blood sample. The resulting chosen material will then be used to design a continuous system for measuring glucose for measured hemodynamic parameters. The measurement is designed as a continuous, prospective use in a catheter with the aim of measuring hemodynamic parameters in any part of the bloodstream. If another enzyme or chemical is used, the system can be used to analyze other reagents if the reaction would result in the formation of free ions.

## Figures and Tables

**Table 1:** Electric characteristics of tested materials at a temperature of 20 degrees Celsius [8].

	Electrical Resistivity	Electrical Conductivity
Material	$10^{-6}\Omega .m$	$\mathrm{MS}.m^{-1}$
Ag	0.016	61.5
Au	0.023	43.5
Cu	0.018	56.2

**Table 2:** Correlation table of Ag/AgCl electrode.

Concentration	Without Enzyme	With Enzyme
[mM]	Arithmetic Mean	Arithmetic Mean
6	0.30	0.44
12	0.42	0.62
20	0.63	0.84
25	0.87	0.99
**Correlation [-]**	0.984034157	0.995836775

**Table 3:** Correlation table of Cu electrode.

Concentration	Without Enzyme	With Enzyme
[mM]	Arithmetic Mean	Arithmetic Mean
6	0.16	0.37
12	0.19	0.43
20	0.27	0.57
25	0.37	0.59
**Correlation [-]**	0.96806106	0.983258322

**Table 4:** Correlation table of Au electrode.

Concentration	Without Enzyme	With Enzyme
[mM]	Arithmetic Mean	Arithmetic Mean
6	0.23	0.30
12	0.29	0.42
20	0.36	0.51
25	0.46	0.61
**Correlation [-]**	0.983292897	0.993248592

**Table 5:** Normality test—*p*-values for concentration of 6 mM.

	Concentration 6 mM	Concentration 6 mM
Material	Without Enzyme	With Enzyme
Ag/AgCl	0.7975	0.5725
Au	0.3523	0.1150
Cu	0.4991	0.6502

**Table 6:** Normality test—*p*-values for concentration of 12 mM.

	Concentration 12 mM	Concentration 12 mM
Material	Without Enzyme	With Enzyme
Ag/AgCl	0.6774	0.8308
Au	0.3345	0.2631
Cu	0.1743	0.8552

**Table 7:** Normality test—*p*-values for concentration of 20 mM.

	Concentration 20 mM	Concentration 20 mM
Material	Without Enzyme	With Enzyme
Ag/AgCl	0.1519	0.7273
Au	0.0768	0.1094
Cu	0.1163	0.8000

**Table 8:** Normality test—*p*-values for concentration of 25 mM.

	Concentration 25 mM	Concentration 25 mM
Material	Without Enzyme	With Enzyme
Ag/AgCl	0.4311	0.2203
Au	0.8652	0.7900
Cu	0.7251	0.3311

**Table 9:** Bartlett’s test.

Concentration	6 mM	12 mM	20 mM	25 mM
without enzyme	0.2652	0.9279	0.2665	0.9303
with enzyme	0.4837	0.6658	0.5953	0.2791
